# A Multimodal Quality Improvement Approach to Reduce Sevoflurane Consumption and Greenhouse Gas Emissions in an Academic Health System

**DOI:** 10.7759/cureus.101430

**Published:** 2026-01-13

**Authors:** Peter Harper, Herodotos Ellinas, Larry Lindenbaum, Neel Kapoor, Abdinoor M Abdi, Nevin S Gupta, Carter S McCauley, Riley A Dougherty, Abdou W Manjang, Vanessa Moll

**Affiliations:** 1 Family Medicine, University of Minnesota School of Medicine, Minneapolis, USA; 2 Anesthesiology, University of Minnesota School of Medicine, Minneapolis, USA

**Keywords:** anesthesiology, greenhouse gas emissions, low-flow anesthesia, perioperative outcomes, quality improvement, sevoflurane, sustainability

## Abstract

Introduction

Volatile anesthetics are major contributors to healthcare-related greenhouse gas (GHG) emissions. After eliminating desflurane from our formulary, we implemented a multimodal quality improvement (QI) initiative to reduce sevoflurane use by adopting low fresh gas flow (FGF) practices.

Methods

This QI project was conducted at two campuses of the University of Minnesota Medical Center (41 operating rooms, ~1,400 cases/month). Interventions between May 2024 and February 2025 included provider education, standardized workflows, monthly data feedback, and anelectronic health record (EHR) best practice alert (BPA) prompting lower FGF use. FGF metrics were derived from intraoperative data with specific inclusion and exclusion criteria, while sevoflurane volume and cost data were obtained directly from SlicerDicer and pharmacy purchase records. Outcomes were analyzed using control charts and two-sample t-tests comparing pre-intervention (January 2023-April 2024) and post-intervention (May 2024-June 2025) periods.

Results

A total of 42,416 general anesthetic cases using sevoflurane met the inclusion criteria. The proportion of cases with average FGF < 2 L/min during the maintenance phase increased from 18.2% to 55.0% (3,886/21,122; 10,323/18,775) (p < 0.001), while those never below 2 L/min decreased from 52.7% to 14.8% (11,128/21,122; 2,812/18,775) (p < 0.001). Mean monthly sevoflurane use declined from 9,785 L to 7,475 L, corresponding to an annual reduction of 35,277 L (approximately 45,000 kg CO₂e) and a net cost savings of approximately $38,000.

Conclusions

At a single academic institution, a multimodal, data-driven QI initiative integrating staff education, workflow optimization, the EHR, and low-flow anesthesia significantly reduced sevoflurane consumption, resulting in net cost savings and a positive environmental impact.

## Introduction

Healthcare is a significant contributor to global greenhouse gas (GHG) emissions, with the United States healthcare sector accounting for an estimated 8-10% of national emissions [1,2]. Within hospitals, operating rooms (ORs) are disproportionately resource-intensive, generating 20-30% of total hospital waste and consuming up to six times more energy than other areas [3]. Among perioperative services, anesthesiology contributes substantially to the environmental footprint through the use of volatile anesthetics, such as sevoflurane, desflurane, and nitrous oxide, which are agents with high global warming potentials (GWPs) and long atmospheric lifespans [4,5]. Volatile anesthetics can account for 5% of emissions from hospitals and 3% of total national health care emissions [6]. Therefore, multiple professional organizations have issued position statements calling for more sustainable perioperative practices [7,8].

Evidence-based strategies to reduce anesthetic-related emissions include eliminating desflurane and nitrous oxide, low flow anesthesia (LFA), minimizing unnecessary material waste, and increasing the adoption of regional anesthesia or total intravenous anesthesia (TIVA) [9,10]. Inhaled anesthetics, especially sevoflurane, remain the predominant choice for general anesthesia in the US. European anesthesiologists have long practiced low-flow anesthesia between 0.5 and 1.0 L/min safely and routinely. In contrast, US clinicians historically avoided very low fresh gas flows (FGF) due to manufacturer warnings about potential renal toxicity from compound A formation with older CO₂ absorbents. Reflecting updated evidence and modern absorber technology, the American Society of Anesthesiologists (ASA) released an official Statement on the Use of Low Gas Flows for Sevoflurane in October 2023 [11], affirming the safety and environmental importance of low-flow practices. Thus, reducing sevoflurane FGF during maintenance represents a high-impact, immediately actionable intervention that is clinically safe and reduces volatile agent use by up to 75% compared with traditional high-flow techniques [10,12]. 

Therefore, after eliminating desflurane from the formulary, our department implemented a multimodal quality improvement (QI) initiative aimed at further reducing sevoflurane-related emissions. This initiative integrated education, real-time electronic healthcare record (EHR) alerts, data feedback mechanisms, and standard work to promote the adoption of reduced FGF. Our primary goal was to increase the proportion of sevoflurane general anesthetic cases conducted with FGF < 2 L/min in the maintenance phase from 18% to 60%, with a secondary goal to decrease overall sevoflurane consumption by 30%.

## Materials and methods

Study design and setting

The QI project was conducted at the University of Minnesota Medical Center (UMMC), a part of a large US academic, multi-specialty healthcare system in Minnesota. The intervention was implemented at two UMMC locations, comprising 41 operating rooms with about 1,400 cases/month, and over 150 anesthesiology residents, certified registered nurse anesthetists (CRNAs), and attending anesthesiologists. Data were extracted from the EHR (EPIC Systems, Verona, WI). This report adheres to the Standards for QUality Improvement Reporting Excellence (SQUIRE 2.0) guidelines for quality improvement reporting [13]. The University of Minnesota Institutional Review Board reviewed the project and determined that it did not constitute human research. 

Interventions

**Figure 1:**
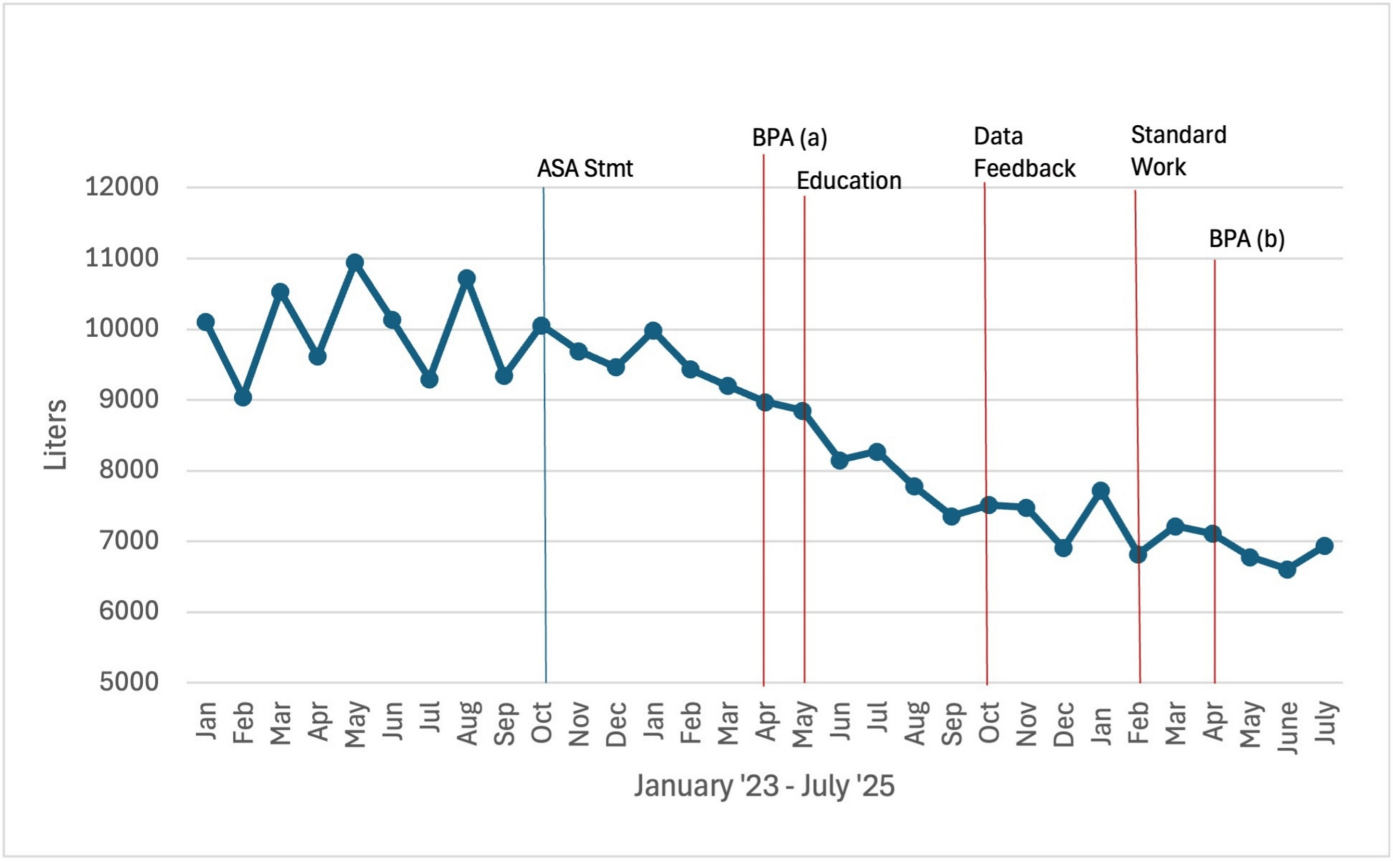
Timeline of interventions and total volume of sevoflurane used per month The red lines depict the start of continuous interventions. ASA Stmt - the American Society of Anesthesiologists Statement on the Use of Low Gas Flows for Sevoflurane in October 2023; BPA – Best Practice Alert (a) initial setting alerted when fresh gas flows (FGF) above 2.2 L/min and (b) changed to alert when FGF above 2.0 L/min

Between May 2024 and February 2025, a series of interventions were implemented sequentially (Figure 1).

Education

Departmental grand rounds, resident didactics, and anesthesia staff meetings introduced the 2023 ASA statement supporting low-flow sevoflurane use and reviewed environmental and cost impacts of volatile anesthetics. There was an initial 45-minute Grand Rounds presentation on reducing FGF. This was followed by monthly 10-minute updates at Quality/Safety meetings on an ongoing basis. Importantly, sessions also incorporated instructions on how to perform low-flow anesthesia. Faculty, residents, and CRNAs were encouraged to participate in the Anesthesia Patient Safety Foundation (APSF) Low-Flow Anesthesia course [14] and the use of the Gas Man® simulation software [15] to model anesthetic uptake, delivery, and wash-in/wash-out kinetics under different flow conditions. 

Real-time Best Practice Alert (BPA) in EPIC

A BPA was adjusted from an EPIC foundation build [16] to alert when average FGF rates exceeded 2.0 L/min when using sevoflurane. The original BPA went live in April 2024 and alerted when FGF was above 2.2 L/min, but was adjusted to the lower value of less than 2.0 L/min in April 2025.

Data Feedback

Before sharing performance data publicly, the project team developed and internally validated an automated FGF < 2 L/min metric using EHR data. Validation was performed by comparing electronically extracted values with manually reviewed anesthesia records by two anesthesiologists to confirm the accuracy of FGF capture and maintenance-phase timestamps. Once validated, the metric was used to generate aggregate performance dashboards starting in September 2024. Beginning in October 2024, monthly departmental feedback reports displayed trends in average FGF < 2 L/min, facilitating discussion, peer comparison, and reinforcement of best practices. Metrics were also presented monthly at Grand Rounds and faculty meetings.

Standard Work

Using Lean methodology, an interdisciplinary team developed “standard work” to address inconsistent FGF practices, based on evidence-based literature and clinicians’ practical experience, and disseminated this standard work across the department in February 2025.

Outcome measures

The intervention was monitored using three measures that were collected and graphed as monthly control charts.

1. Percentage of Cases With Average FGF < 2 L/min During Maintenance Phase

FGF data were automatically captured from the anesthesia machine interface and recorded in the EHR at one-minute intervals. The maintenance phase was defined as 10 minutes after airway placement until 10 minutes before extubation. Inclusion criteria were all general anesthetics defined by the presence of an endotracheal tube or laryngeal mask airway, or an end-tidal volatile agent concentration > 0.3%, or nitrous oxide use for > 5 minutes. Exclusion criteria included cases ≤ 30 minutes, out-of-OR procedures, bronchoscopy or laryngoscopy, and TIVA. For each case, the average FGF during maintenance was calculated, and the monthly proportion of cases meeting the <2 L/min threshold was plotted as a P-control chart.

2. Percentage of Cases that Never Turned Down FGF < 2 L/min During Maintenance Phase

Using the same dataset, one-minute interval data were analyzed to determine whether the FGF was ever reduced below 2 L/min during maintenance. Cases with no recorded value below this threshold were classified as “never turned down.” The monthly proportion was plotted as a P-control chart.

3. Total Sevoflurane Volume/Month

Total sevoflurane volume use was collected from Slicer Dicer in EPIC for each case. Because SlicerDicer does not allow phase-specific data extraction, total intraoperative sevoflurane volume, rather than maintenance-phase volume alone, was analyzed to assess the overall impact on agent consumption and GHG emissions. The accuracy of EPIC volume data was validated by comparison to sevoflurane purchase data. Monthly sevoflurane total volume was plotted on an I-control chart. In addition, the average sevoflurane volume/case and average sevoflurane volume/minute were calculated to ensure that changes in total sevoflurane use were NOT due to fewer cases or shorter surgeries.

Cost and emissions calculations

Sevoflurane costs were estimated by the number of sevoflurane bottles used annually based on the sevoflurane volume used. Purchase data were collected and compared to estimates. Although similar, purchase data slightly underestimated actual savings, likely due to the inventory methodology. The annual sevoflurane cost was estimated by dividing the total volume (L/year) by 42.6 L/bottle and multiplying by $60.82 per bottle (cost provided by pharmacy). A balancing measure of CO2 absorber utilization was monitored to assess potential changes in the circuit with low flow FGF. CO₂-absorber utilization and costs were obtained from purchasing records for 2023-2025, with 2025 data annualized from January to June.

GHG emissions were calculated from the number of bottles of sevoflurane saved using the formula: Number of bottles x bottle volume x density x GWP = total kgCO2e [Bottle Volume - 0.25 liters (250ml), Density - 1.52, GWP - 144]. GHG equivalencies were expressed using the U.S. EPA Greenhouse Gas Equivalencies Calculator [17].

Statistical analysis

Monthly total sevoflurane volume was analyzed using I-control charts, and the two proportion-based measures using P-control charts. Pre-intervention (January 2023 - April 2024) and post-intervention (May 2024 - June 2025) means were compared with two-sample t-tests. Analyses were performed using Minitab Statistical Software, version 21 (Minitab LLC, State College, PA). Statistical significance was defined as p < 0.05. Because sevoflurane volume and cost data were extracted directly from SlicerDicer, which does not allow filtering by case duration or anesthetic type, these analyses included all sevoflurane-containing cases without additional exclusions. In contrast, FGF metrics were derived from electronically captured intraoperative data with specific inclusion and exclusion criteria, leading to slight differences in case counts.

## Results

A total of 42,416 sevoflurane general anesthetics met the inclusion criteria across the 30-month study period, with comparable monthly case volumes between pre-intervention (1,412 ± 92 cases/month) and post-intervention (1,415 ± 87 cases/month; p = 0.89) (Table 1).

**Table 1:** Sevoflurane utilization pre- and post-intervention ^a^Average liters of sevoflurane used per case, calculated from total monthly sevoflurane volume divided by total number of cases. ^b^Average liters of sevoflurane used per minute, calculated from total monthly sevoflurane volume divided by total anesthesia time per case Statistical test: two-sample T-test Diff.: difference (pre-/post-intervention); SD: standard deviation

	Pre-intervention	Post-intervention	Diff.	T- statistic	P-value
Months	16	14			
Total cases, n	22,593	19,823			
Cases/month, mean ± SD	1,412 ± 87.9	1,415 ± 56.0	3	0.15	0.886
Vol/month, mean ± SD	9,785 ± 598	7,475 ± 637	-2310	-10.2	<0.001
L/case - average^a^, mean ± SD	6.94 ± 0.37	5.28 ± 0.335	-1.66	-12.93	<0.001
L/min - average^b^, mean ± SD	0.051 ± 0.0018	0.040 ± 0.0023	-0.011	-14.52	<0.00

Primary outcome: FGF metrics

**Figure 2:**
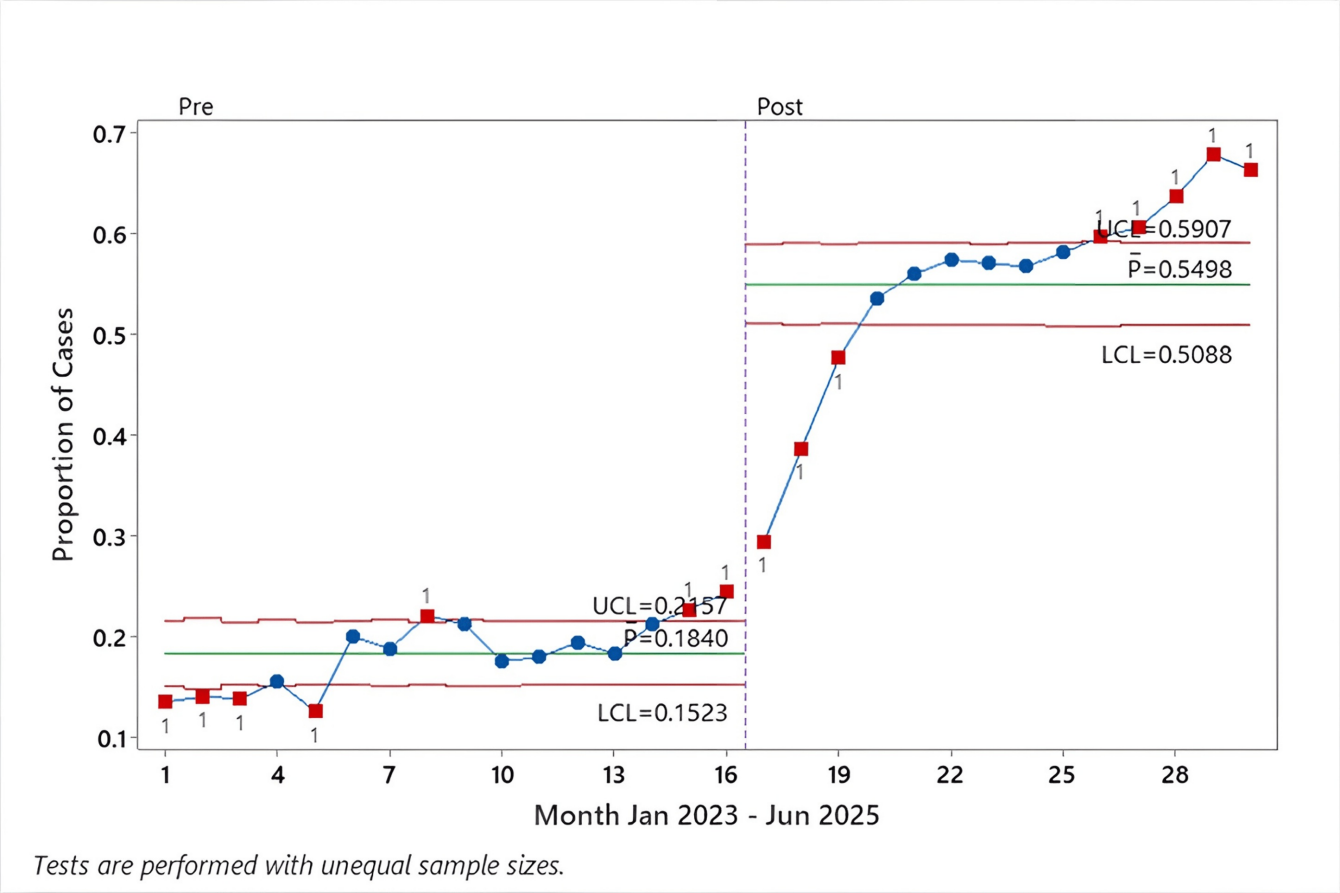
P-control chart of the proportion of cases with average FGF < 2 L/min during the maintenance phase (January 2023 - June 2025), segmented by intervention period The proportion of anesthesia cases that averaged an FGF of less than 2 L/min is plotted monthly from January 2023 to June 2025. The dashed vertical line marks the start of the post-intervention period (May 2024). Each point represents a monthly proportion, with special-cause variation indicated by red squares. The green horizontal lines represent the center lines/mean proportion, while the red horizontal lines represent the UCL and LCL FGF: fresh gas flow; UCL: upper control limit; LCL: lower control limit

**Figure 3:**
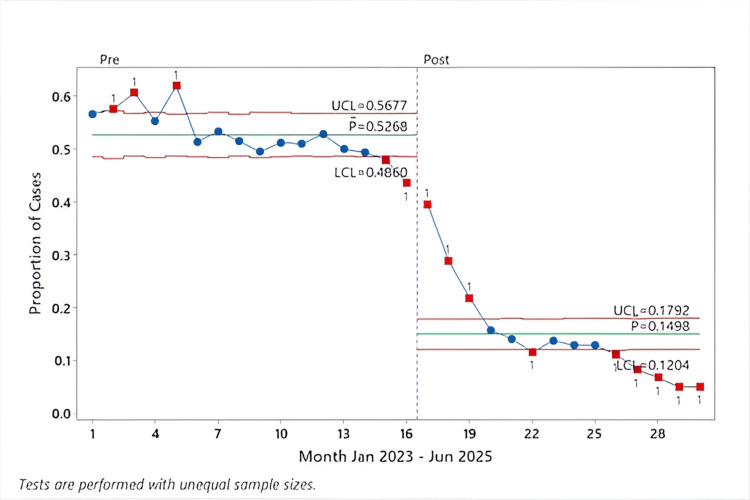
P-control chart of the proportion of cases with FGF never below < 2 L/min (January 2023-June 2025), segmented by intervention periods The proportion of anesthesia cases in which FGF never reduced to less than 2 L/min is plotted monthly from January 2023 to June 2025. The dashed vertical line marks the start of the post-intervention period (May 2024). Each point represents a monthly proportion, with special-cause variation indicated by red squares. The green horizontal lines represent the center lines/mean proportion, while the red horizontal lines represent the UCL and LCL FGF: fresh gas flow; UCL: upper control limit; LCL: lower control limit

The proportion of cases with average FGF < 2 L/min during the maintenance phase increased from 18.2% to 55.0% (3886/21,122; 10,323/18,775) (p < 0.001) (Table 2, Figure 2). Conversely, the proportion of cases with FGF never below < 2 L/min decreased from 52.7% to 14.8% (11,128/21,122; 2812/18,775) (p < 0.001), demonstrating substantial behavioral change across the department (Table 2, Figure 3).

**Table 2:** FGF metrics pre- and post-intervention ^a^Percentage of OR cases that averaged an FGF of less than 2 liters per minute. ^b^Percentage of OR cases that never averaged an FGF of less than 2 liters per minute Statistical test: two-sample T-test FGF: fresh gas flow; Diff.: difference (pre-/post-intervention); SD: standard deviation

	Pre-intervention	Post-intervention	Diff	T-statistic	p-value
Total cases, n	21,122	18,772			
Cases/month, mean ± SD	1,320 ± 89.7	1,341 ± 50.9	21	0.79	0.437
% cases ave FGF < 2 L/min^a^, mean ± SD	18.2% ± 3.6%	55.0% ± 10.5%	36.80%	12.52	<0.001
% cases never FGF < 2 L/min^b^, mean ± SD	52.7% ± 4.7%	14.8% ± 9.6%	-37.90%	-13.45	<0.001

Secondary outcome: sevoflurane utilization

**Figure 4:**
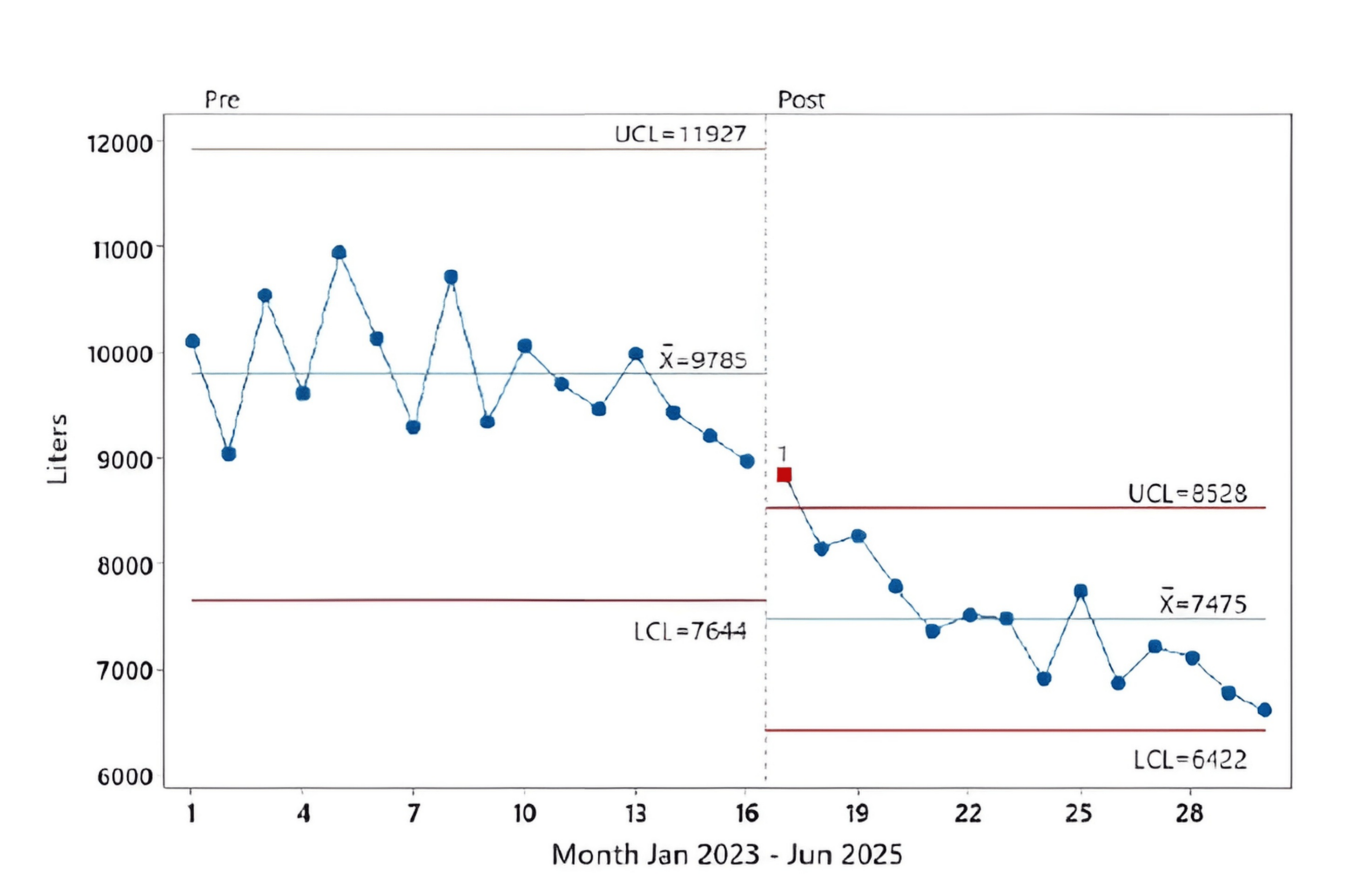
I-control chart of monthly total sevoflurane volume (L/month, January 2023 – June 2025), segmented by intervention period This figure shows monthly total sevoflurane volume (L/month) plotted on an Individuals (I) control chart from January 2023 to June 2025. The dashed vertical line marks the start of the post-intervention period (May 2024). The centerline (X) represents the mean monthly volume for each period. The red horizontal lines represent the UCL and LCL. The post-intervention period shows a sustained downward trend in monthly sevoflurane use UCL: upper control limit; LCL: lower control limit

Total sevoflurane use declined from 9,785 L/month to 7,475 L/month (difference 2,310 L/month, p < 0.001) (Table 1, Figure 4). Mean sevoflurane consumption per case decreased from 6.94 L/ case to 5.28 L/case (diff 1.665; p < 0.001), and per anesthesia-minute from 0.051 L/min to 0.040 L/min (diff 0.011; p < 0.001) (Table 1).

Cost and environmental impact

Annualized sevoflurane usage was reduced by 35,277 L/year, corresponding to an estimated reduction of 45,308 kg/CO₂e year in greenhouse gas emissions. Emissions from CO_2_-absorbers were not calculated due to technical challenges. The associated cost savings in sevoflurane totaled $50,359 per year, partially offset by a $12,000 per year increase in CO₂-absorber costs, yielding a net annual savings of approximately $38,000.

## Discussion

Our QI initiative demonstrated that a structured, multimodal approach can meaningfully reduce anesthetic-related GHG emissions. The intervention, which included education, introduction of sevoflurane BPA, data-driven feedback, and standardized workflows, led to a statistically significant increase in the proportion of anesthetics conducted with FGF < 2 L/min during the maintenance phase. In parallel, the proportion of cases with FGF never below < 2 L/min dropped with sustained improvement to only 5% by project end. Although the targets were not fully met, the results represent a statistically significant and clinically meaningful change. These findings confirm that educational and workflow-based interventions can change long-standing clinical habits and achieve substantial sustainability gains. The change in sevoflurane consumption translated into a reduction in annual sevoflurane utilization, with a significant environmental impact based on CO₂-equivalent calculations. This aligns with prior work showing that lowering FGF decreases volatile agent use and emissions by 50-75% without affecting patient safety [10,18]. In addition, our institution realized approximately US $38,000 in annual net savings, highlighting the dual environmental and financial benefits of LFA.

A central component of our educational campaign was addressing outdated concerns about sevoflurane nephrotoxicity due to exposure to “compound A.” Those data were derived from rat studies using obsolete soda-lime absorbers containing strong bases [19], which are no longer in use. Modern CO₂ absorbers effectively reduce the formation of compound A, and no evidence of clinically relevant renal injury has been demonstrated in humans [20]. Consequently, European regulators never imposed minimum FGF restrictions, and LFA has been widely and safely adopted there. In contrast, US practice was historically constrained by manufacturer labeling that recommended flows greater than 2 L/min. The ASA removed this barrier with its Statement on the Use of Low Gas Flows for Sevoflurane (October 2023), confirming that sevoflurane is safe even at FGF below 1 L/min and encouraging environmentally responsible practice [11].

Our success likely stemmed from combining education and standard work with visual prompts (BPA) and data-driven feedback. We embedded sustainability into clinical culture through electronic best practice alert reminders and a sustainability dashboard integrated within the anesthesia information management systems (EPIC). Studies show that such real-time decision-support alerts can cut volatile anesthetic waste and FGF rates by 10-30% [21,22].

Broader implications for health-system sustainability

LFA aligns with institutional and national decarbonization targets, including the 2022 US Department of Health and Human Services Health Sector Climate Pledge, global Net-Zero Healthcare initiatives [23], and the United Nations Sustainable Development Goals [24]. Because operating rooms are among the most energy-intensive hospital environments, consuming 3-6 × the energy of standard wards [3], anesthesia departments are uniquely positioned to lead measurable emission reductions through behavioral interventions that do not require significant capital investment. The estimated 45,000 kg CO₂e reduction achieved by this initiative is equivalent to eliminating the annual emissions of 114,595 miles driven by an average gasoline-powered car [17]. This illustrates that seemingly small clinical choices scale to meaningful system-level impact. 

Safety, quality, and occupational health

Beyond environmental benefits, lowering FGF reduces occupational exposure to halogenated agents, substances associated with reproductive and neurological risks in chronic exposure studies [25]. Additionally, sevoflurane belongs to per- and polyfluoroalkyl substances (PFAS), now recognized as persistent contaminants [26]. The dual benefit of protecting both patient and provider safety strengthens the ethical imperative for implementation.

Implementation and cultural considerations

Despite compelling evidence, LFA adoption remains variable. Barriers include cultural inertia and a lack of feedback mechanisms. Embedding environmental sustainability metrics into professional quality dashboards, maintenance phase checklists, and resident evaluations may enhance accountability. Integrating sustainability into continuing medical education and simulation curricula could help further normalize climate-conscious practices among all anesthesia providers.

Limitations and future directions

Limitations of our initiative include dependence on voluntary attendance at grand-round sessions and the absence of individualized feedback during the study. Additionally, this project was conducted within a single academic health system, which may limit its generalizability to community or resource-limited settings. Minor discrepancies in case numbers between Tables 1 and 2 reflect differences in data sources and inclusion criteria. FGF metrics were restricted to cases meeting predefined analytic criteria, whereas sevoflurane volume and cost data were drawn directly from SlicerDicer without phase-specific exclusions. These variations do not affect the overall interpretation of trends or conclusions.

Future work should assess the durability of behavior change, explore provider-specific determinants of adherence, and quantify long-term cost and emissions savings. Incorporating individualized dashboards, benchmarking against peers, and joining quality initiatives such as the Multicenter Perioperative Outcomes Group (MPOG) [27] represent promising next steps. Subsequent efforts could also extend beyond the current <2 L/min target by exploring low flow (typically 0.5-1 L/min) and metabolic FGF, which delivers fresh gas at rates approximating the patient’s oxygen consumption (typically 0.25-0.5 L/min in adults). This transition to metabolic FGF could be significantly enhanced by collaborating with anesthesia machine vendors. A key opportunity lies in advocating for the introduction of automated end-tidal concentration control systems into the US market, a technology that is already available in other countries [28]. These closed-loop systems enable the provider to set the desired end-tidal oxygen and anesthetic agent targets; the machine then automatically adjusts the FGF and vaporizer output (often to minimal or metabolic levels) to precisely and efficiently maintain these targets.

Adopting such technology could standardize low-flow delivery, minimize waste, and substantially reduce volatile anesthetic consumption and greenhouse gas emissions. Lastly, healthcare pollution is often attributed to "end-of-life" emissions from patient exhalation, underestimating the total climate impact by omitting the significant "cradle-to-grave" emissions from the manufacturing process itself [29]. Recent life cycle assessments highlight that fugitive emissions during production can be substantial, as manufacturers are not universally required to implement vapor reclamation or thermal oxidation technologies, which are critical for abating emissions at the source [6]. It should be noted that this study did not attempt to do a full “cradle-to-grave” analysis, making total emission calculations incomplete.

## Conclusions

This QI initiative demonstrates that combining provider education, BPAs, data-driven feedback, and standardized workflows can significantly decrease volatile anesthetic use and associated greenhouse-gas emissions. These results support the 2023 ASA guidance on low-flow sevoflurane use and reinforce anesthesiology’s leadership role in reducing healthcare’s climate footprint. LFA is a safe, cost-effective, and environmentally responsible practice that should be integrated into routine perioperative care through continued education, workflow optimization, and electronic feedback systems.
